# Early-stage corneal toxicity secondary to high-dose systemic cytarabine: a case report

**DOI:** 10.1186/s12886-023-02834-3

**Published:** 2023-03-06

**Authors:** Tsuyoshi Mito, Shun Takeda, Hisanori Miyashita, Hiroshi Sasaki

**Affiliations:** Department of Ophthalmology, Kanazawa Medical University, 1-1 Daigaku, Uchinada, Kahoku, Ishikawa 920-0293 Japan

**Keywords:** Cytarabine, Corneal toxicity, Corneal epithelial microcysts, Leukemia, Case report

## Abstract

**Background:**

High-dose systemic cytarabine chemotherapy may cause fine corneal opacities and refractile microcysts, which are densely distributed in the center of the cornea. Most previous case reports on microcysts have been those following complaints of subjective symptoms, and the findings at the initial stage of development and time-course changes are still unknown. This report aims to clarify the time-course changes of microcysts using slit-lamp photomicrographs.

**Case presentation:**

A 35-year-old woman who was treated with high-dose systemic cytarabine therapy (3 courses of 2 g/m^2^ every 12 h for 5 days) for acute myeloid leukemia and presented with subjective symptoms, such as bilateral conjunctival injection, photophobia, and blurred vision, on the 7^th^ day of treatment in both the first two courses. Anterior segment findings by slit-lamp microscopy revealed microcysts densely distributed in the central region of the corneal epithelium. In both courses, microcysts disappeared within 2–3 weeks upon prophylactic steroid instillation. In the 3^rd^ course, daily ophthalmic examinations were conducted from the start of the treatment, and on the 5^th^ day without subjective symptoms, the microcysts in the corneal epithelium appeared evenly and sparsely distributed throughout the cornea except for the corneal limbus. Thereafter, the microcysts accumulated towards the center of the cornea and disappeared gradually. The change from low-dose to full-strength steroid instillation immediately following the occurrence of microcysts in the 3^rd^ course resulted in the peak finding being the mildest compared to that in the past two courses.

**Conclusions:**

Our case report revealed that microcysts appeared scattered throughout the cornea before the appearance of subjective symptoms and then accumulated in the center and disappeared. A detailed examination is necessary to detect early changes in microcyst development resulting in prompt and appropriate treatment.

## Background

Cytarabine, an antimetabolite malignant tumor drug, is an essential drug for the treatment of hematopoietic tumors, such as acute myeloid leukemia. High-dose systemic cytarabine chemotherapy is several tens of times higher than the usual dose; thus, it may cause systemic side effects, such as central nervous system toxicity and myelosuppression [1]. Considering the ophthalmic side effects, it was reported that 85% of the patients reported eye problems, such as conjunctivitis, conjunctival injection, lacrimation, and photophobia [2]. In addition, fine corneal opacities and refractile microcysts, which are densely distributed in the center of the cornea and are reported as a characteristic ocular finding in the corneal epithelium, are likely to occur based on the dose or duration of cytarabine administration [3, 4]. However, most previous case reports on microcysts have been those following complaints of subjective symptoms, and the findings at the initial stage of development are still unknown. Moreover, there have been only a few reports detailing time-course changes, since ocular symptoms coincide with the systemic side effects attributed to high-dose cytarabine therapy, and it is often difficult to perform an ophthalmic examination in such situations.

We report the initial onset of microcysts in the corneal epithelium before subjective symptoms and their subsequent course using slit-lamp photomicrographs.

## Case presentation

**Fig. 1:**
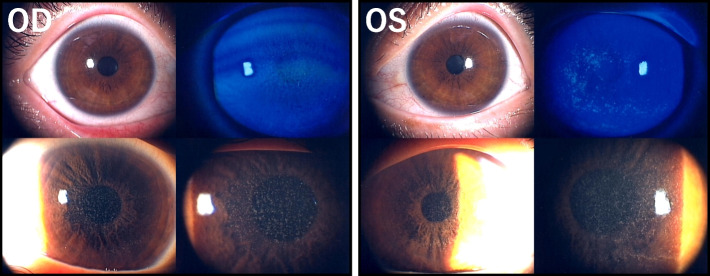
Slit-lamp photomicrographs of microcysts caused by high-dose systemic cytarabine therapy in the 1^st^ course. On the 8^th^ day after the start of cytarabine therapy, bilateral mild conjunctival hyperemia, ciliary injection, and superficial punctate keratopathy were observed. The transillumination images showed fine corneal opacities and refractile microcysts densely distributed in the center of the corneal epithelium; however, no inflammation in the anterior chamber was observed. OD, right eye; OS, left eye

A 35-year-old Japanese woman was diagnosed with acute myeloid leukemia and was treated with a combination of idarubicin and cytarabine as remission induction therapy. High-dose systemic cytarabine therapy was subsequently scheduled for 3 courses, every 2 months, as consolidation therapy. A single course comprised cytarabine administration at 2 g/m^2^ 10 times every 12 h for 5 days. From the morning of the 7^th^ day following the start of the first course, she presented with eye pain, lacrimation, photophobia, and blurred vision in the left eye, and was referred to our department of ophthalmology the next day. At the first examination, her corrected visual acuity (VA) was 20/25 in the right eye and 20/40 in the left eye. Considering the anterior segment findings, the slit-lamp microscopy revealed bilateral mild conjunctival hyperemia, mild superficial punctate keratopathy, and granular microcysts densely distributed in the central region of the corneal epithelium, which were slightly more in the left eye (Fig. 1). There was no inflammation in the anterior chamber, no abnormalities upon corneal endothelium examination, no abnormal findings in the cornea upon anterior segment optical coherence tomography (CASIA2; Tomey Corporation, Nagoya, Japan), and no obvious abnormalities were detected in the optic media and fundus.

To prevent corneal epithelial damage attributed to cytarabine, the patient was administered 0.02% fluorometholone eye drops and artificial tears four times a day by the oncologist at the same time as the start of instillation treatment; however, after the ophthalmology referral, 0.1% fluorometholone eye drops and artificial tears were administered 6 times a day, and ofloxacin eye ointment was applied once before sleep. On the 11^th^ day following the start of the treatment, the patient was isolated in a sterile room owing to cytopenia following severe myelosuppression, which is a side effect of high-dose systemic cytarabine therapy; therefore, we conducted a bedside examination. Thereafter, the microcyst gradually decreased, subjective symptoms disappeared on the 14^th^ day, the microcyst disappeared completely on the 28^th^ day, and the corrected VA finally recovered to 20/20 in both eyes.

At the start of the second course, 0.1% fluorometholone eye drops and artificial tears were prophylactically administered four times a day; however, she reported eye pain, lacrimation, and blurred vision 7 days following the start of treatment, similar to the first course. She underwent reexamination at our department, and densely distributed microcysts in the center of the cornea, were detected in both eyes. The 0.1% fluorometholone instillation was increased to 6 times a day, and the subjective symptoms improved on the 9^th^ day; the microcysts disappeared on the 17^th^ day.

**Fig. 2:**
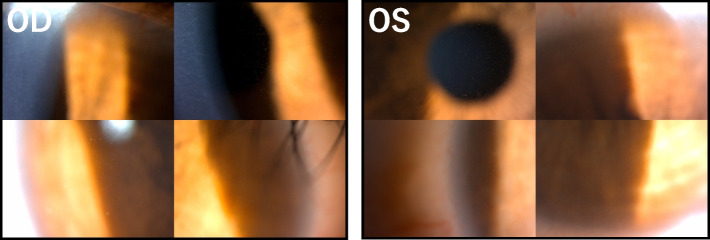
Slit-lamp photomicrographs immediately after the occurrence of microcysts in the 3^rd^ course. On the 5^th^ day after the start of cytarabine therapy, bilateral microcysts were evenly and sparsely distributed throughout the cornea except for the corneal limbus. No subjective symptoms were observed at this time. OD, right eye; OS, left eye

**Fig. 3:**
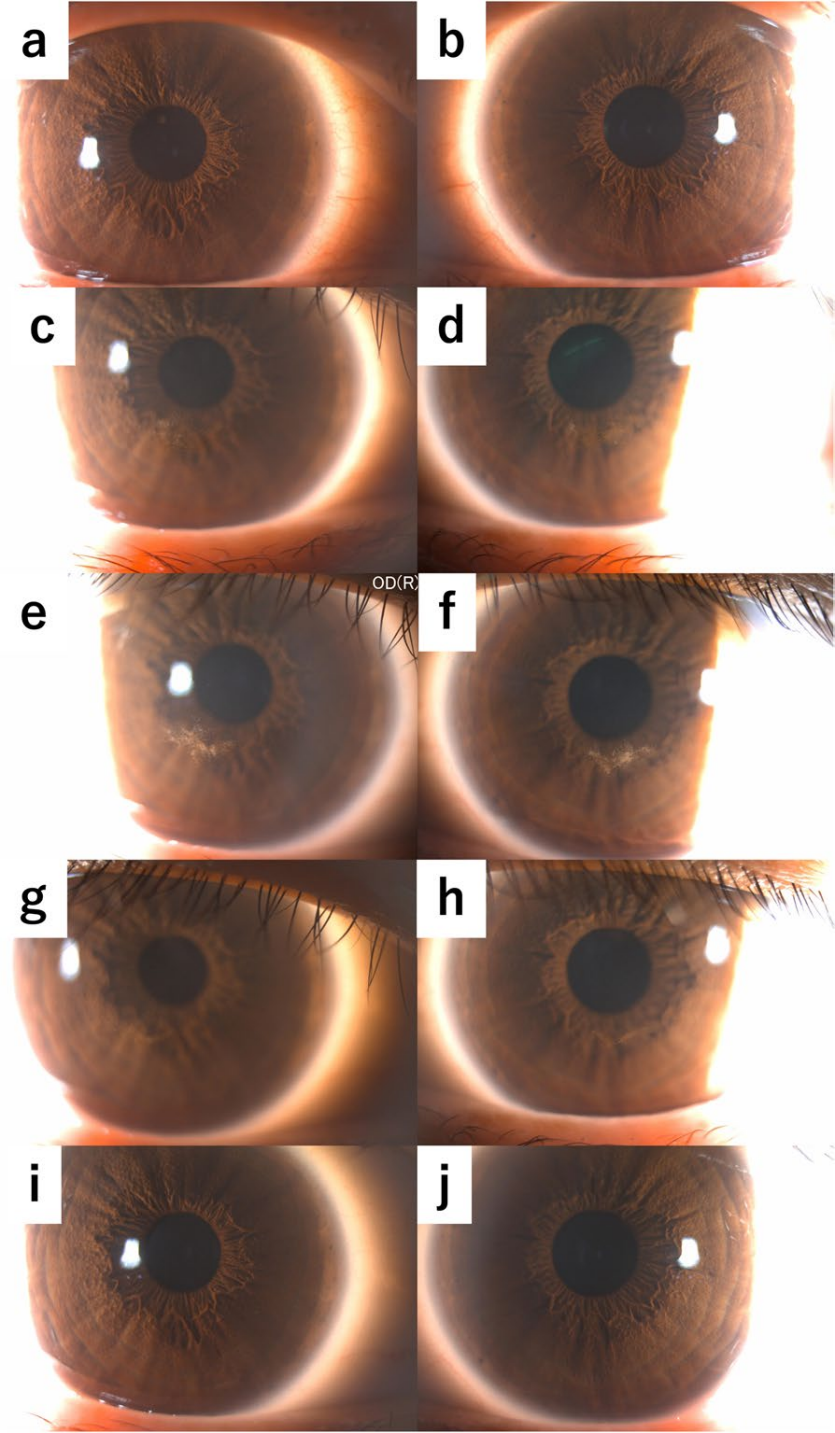
Time-course changes of microcysts in the 3^rd^ course. a, c, e, g, i, right eye; b, d, f, h, j, left eye. On the 5^th^ day (**a**, **b**) of the start of cytarabine therapy, eye drops were changed from 0.1% fluorometholone to 0.1% betamethasone immediately following the onset of microcysts. On the 6^th^ day (**c**, **d**), the microcysts began to accumulate slightly below the center of the cornea. On the 7^th^ day (**e**, **f**), microcyst accumulation in the center of the cornea was at the peak. There were no microcysts in the corneal limbus. The patient complained of subjective symptoms at this time. On the 8^th^ day (**g**, **h**), the microcysts began to disappear gradually. It was impossible to record the corneal lesions as photomicrographs from the next day since the patient was isolated in a sterile room owing to the cytopenia. On the 21^st^ day (**i**, **j**), microcysts completely disappeared and no ophthalmologic residual disorders were observed

A high possibility of the microcyst recurrence with the start of the third course was considered, and the patient underwent daily ophthalmic examinations from the start date of the treatment. No abnormal findings were detected in the cornea until the 4^th^ day; however, on the morning of the 5^th^ day, the fluorescein-negative microcysts, which were evenly and sparsely distributed over the entire cornea except for the width of approximately 1–2 mm in the corneal limbus, were observed without subjective symptoms (Fig. 2). Considering the findings of recurrence, 0.1% fluorometholone instillation, which was administered prophylactically, was changed to 0.1% betamethasone instillation six times a day. On the 6^th^ day, the microcysts accumulated slightly below the center of the cornea, and on the 7^th^ day, the accumulation of microcysts in the center of the cornea peaked; however, the peripheral area of the cornea became transparent and no new microcysts were observed. Similar to the previous two courses, subjective symptoms appeared on the 7^th^ day following the start of treatment; however, they were milder than those at the time of the previous two courses. On the 8^th^ day, the microcysts in the center of the cornea slightly disappeared and only remained in a linear shape (Fig. 3a-j). Thereafter, since the patient was isolated in a sterile room owing to cytopenia, it was impossible to record the corneal lesions by taking photographs. Approximately 1 week later, the microcysts completely disappeared, and the final corrected VA was 20/20 in both eyes; no ophthalmologic residual disorders were observed.

## Discussion and conclusions

To our knowledge, this is the first case to record corneal lesions on slit-lamp photomicrographs depicting the initial onset and time courses of the microcysts induced by high-dose systemic cytarabine therapy in patients with acute myeloid leukemia. Corneal toxicity owing to high-dose systemic cytarabine therapy generally appears on the 5^th^ to 7^th^ day following the start of treatment and then spontaneously disappears in nearly a week following the appearance [3]. Since the onset of corneal toxicity coincides with the worsening of leukemia or systemic side effects, such as severe myelosuppression, performing frequent ophthalmic examinations is challenging and often requires bedside visits. Therefore, there has been no detailed report on the course of microcysts. Previous reports have demonstrated that microcysts were densely distributed in the central region of the cornea [3, 4] however, our daily observations revealed that microcysts had already occurred 1–2 days prior to the development of subjective symptoms, and in the early stages of the disease, microcysts were evenly and sparsely distributed throughout the cornea except for the corneal limbus, and thereafter, they accumulated in the center of the cornea and disappeared gradually.

Corneal toxicity attributed to cytarabine was first recognized in the 1960s; cytarabine ophthalmic preparation, which was a candidate therapeutic agent for herpes keratitis, reportedly caused drug toxicity to the corneal epithelium in vivo [5, 6]. In 1981, Hopen et al*.* reported a case of corneal toxicity in high-dose systemic cytarabine therapy for patients with leukemia and referred to the granular lesion in the corneal epithelium as a “microcyst [3].” In their reports, pathological findings revealed that the degenerated cells, which are a mixture of pyknosis and cytoplasmic fragments, were the main body of the microcyst, and not cystic cavities. The following hypothesis is proposed to explain the occurrence of microcysts in the corneal epithelium. Generally, in the corneal epithelial layer, transient amplifying cells (TACs) differentiate from limbal epithelial stem cells (LESCs) in the corneal limbus and migrate towards the corneal center, undergo intermittent cell division, differentiate into superficial cells, and eventually fall off from the surface layer, thereby maintaining the homeostasis of the corneal epithelium [7]. On the other hand, cytarabine is metabolized to Ara-CTP in the cytoplasm and inhibits DNA polymerase, DNA repair, and RNA synthesis. Since these actions prominently occur in the cells in the S phase (DNA replication phase) of the cell cycle, cells with faster dividing cycles are more likely to be affected [8]. Thus, cytarabine may affect TACs with a short cell cycle compared to LESCs with a long cell cycle in the corneal epithelium [9]. If the LESCs are more susceptible to cytarabine, early-stage microcysts are expected to develop at high density from the corneal limbus; however, in this case, the microcysts were evenly and sparsely distributed throughout the cornea with transparency of the corneal limbus, supporting the hypothesis that the TACs that are widely present in the corneal epithelium are damaged by cytarabine.

Guthoff et al*.* observed the time-course changes of cells damaged by cytarabine in the center of the cornea using in vivo confocal microscopy following subjective symptoms [10]; they observed degenerated cells in the basal epithelial layer on the 1^st^ day, in the basal–apical layer on the 3^rd^ to 4^th^ days, and many degenerated cells in the superficial layer on the 9^th^ to 14^th^ days. These findings suggest that degenerated cells move from the deep layer to the surface layer of the corneal epithelium. In this observation as well, the time-course change of the microcysts scattered throughout the cornea, accumulating at the center of the cornea and disappearing gradually, may be one of the important findings for clarifying the processes from the development of microcysts to their disappearance. Microcysts densely distributed in the central region of the cornea, as described in previous papers, are thought to be the result of degenerated cells after accumulating towards the center for several days owing to inhibited cell synthesis in the corneal epithelial basal cell layer.

The effectiveness of steroid eye drops (1% prednisolone) for corneal toxicity by cytarabine has been previously reported [11, 12]. The suppression of DNA replication by steroids would render corneal epithelial basal cells less susceptible to the effects of cytarabine. Thus, steroids were administered prophylactically in all three courses; however, microcysts at the peak were most severe in the 1^st^ course owing to the administration of the weakest 0.02% fluorometholone instillation. In the 3^rd^ course, the same 0.1% fluorometholone instillation as in the 2^nd^ course was administered prophylactically; however, owing to changing the eye drops to 0.1% betamethasone instillation immediately following the onset of fine microcysts before the development of subjective symptoms, the degree of microcysts at the peak was the mildest and the subjective symptoms were minor. This change would have prevented the injury of the TACs, associated with the remaining course of cytarabine administration over the next few days. Thus, although steroids cannot completely prevent corneal toxicity attributed to cytarabine, it may be possible to suppress the peak of symptoms by increasing the strength of the eye drops and the instillation times.

This case report presents findings from the pre-occurrence to the disappearance of microcysts attributed to high-dose systemic cytarabine therapy. A detailed examination of patients receiving cytarabine therapy without subjective symptoms is necessary to detect early changes in microcyst development resulting in prompt and appropriate treatment, thereby enabling the reduction of corneal toxicity and its concomitant subjective symptoms.

## Data Availability

All data generated or analyzed during this study are included in this published article.

## Abbreviations

LESCs: Limbal epithelial stem cells
TACs: Transient amplifying cells
VA: Visual acuity
